# Feasibility and Preliminary Effects of Adding Percutaneous Electrical Nerve Stimulation to a Pain Education and Exercise Program in Patients with Knee Osteoarthritis: A Pilot Randomized Controlled Trial

**DOI:** 10.3390/jcm15020624

**Published:** 2026-01-13

**Authors:** Leonardo Rodríguez-Lagos, Alberto Arribas-Romano, Sofía Laguarta-Val, Beatriz Serrano García, Daniel Martín-Vera, Angela Menéndez-Torre, Josué Fernández-Carnero

**Affiliations:** 1 Cognitive Neuroscience, Pain and Rehabilitation Research Group (NECODOR), Faculty of Health Sciences, Rey Juan Carlos University, 28922 Alcorcón, Spain; l.rodriguezla.2019@alumnos.urjc.es (L.R.-L.); sofia.laguarta@urjc.es (S.L.-V.); angela.menendez@eug.es (A.M.-T.); josue.fernandez@urjc.es (J.F.-C.); 2 Department of Physical Therapy, Occupational Therapy, Rehabilitation and Physical Medicine, Rey Juan Carlos University, 28922 Alcorcón, Spain; 3 Departamento de Ciencias Médicas Básicas, Facultad de Medicina, Universidad San Pablo-CEU, CEU Universities, Urbanización Montepríncipe, 28660 Boadilla del Monte, Spain; beatriz.serranogarcia@ceu.es; 4 Department of Physiotherapy, Faculty of Medicine, Health and Sports, European University of Madrid, 28670 Villaviciosa de Odón, Spain; daniel.martin2@universidadeuropea.es; 5 Clínica Axium Salud Funcional, 28807 Alcalá de Henares, Spain; 6 Research Group on Exercise Therapy and Functional Rehabilitation, Faculty of Medicine, Health and Sports, European University of Madrid, 28670 Villaviciosa de Odón, Spain; 7 Departamento de Fisioterapia, Universidad de Cantabria, 39300 Torrelavega, Spain; 8 Servicio de Fisioterapia, Vitasana, 33004 Oviedo, Spain; 9 Motion in Brains Research Group, Institute of Neuroscience and Sciences of the Movement (INCIMOV), Centro Superior de Estudios Universitarios La Salle, 28023 Madrid, Spain; 10 Musculoskeletal Pain and Motor Control Research Group, Faculty of Health Sciences, Universidad Europea de Madrid, 28670 Villaviciosa de Odón, Spain; 11 La Paz Hospital Institute for Health Research (IdiPAZ), 28029 Madrid, Spain

**Keywords:** PENS, percutaneous electrical nerve stimulation, knee osteoarthritis, pain sensitization, knee pain

## Abstract

**Objectives**: To estimate the preliminary effects of adding percutaneous electrical nerve stimulation (PENS) to a pain education and exercise program on pain sensitization, function, and psychological factors in patients with knee osteoarthritis (KOA), compared with adding a control transcutaneous electrical nerve stimulation (TENS) intervention or sham PENS. Feasibility, safety, and the success of participant blinding were also evaluated. **Methods**: Thirty patients with KOA were randomly assigned to one of three intervention groups: PENS, control TENS, or sham PENS. All interventions were delivered in addition to a program comprising four pain education sessions and a structured 12-week exercise plan. Primary outcomes included the Western Ontario and McMaster Universities Osteoarthritis Index (WOMAC), Visual Analogue Scale (VAS), Chronic Pain Grading Scale (CPGS), Pressure Pain Threshold (PPT), Conditioned Pain Modulation (CPM), and Temporal Summation of Pain (TSP). Psychological variables were assessed as secondary outcomes. Feasibility outcomes included recruitment and retention rates, adherence, adverse events, and blinding success. **Results**: Significant and consistent improvements over time were observed across the full sample for VAS (Chi^2^ = 13.38; *p* = 0.004), CPGS (Chi^2^ = 15.22; *p* = 0.002), WOMAC (Chi^2^ = 31.44; *p* < 0.001), CPM (Chi^2^ = 8.77; *p* = 0.032) and TSP (Chi^2^ = 53.11; *p* < 0.001) with changes in potential clinical relevance at the within-group level. However, no statistically significant group–time interactions were found for any variable, suggesting no clear differential effects between interventions. Feasibility outcomes were favorable, with high retention and adherence, a low incidence of mild adverse events, and generally adequate participant blinding. **Conclusions**: Within the limits of this small exploratory trial, adding PENS to a pain education and exercise program did not appear to provide additional benefits in pain sensitization, function, or psychological factors beyond those achieved with the multimodal program and sham or control electrical stimulation. Feasibility, safety, and blinding outcomes support the viability of conducting a larger definitive trial.

## 1. Introduction

Knee osteoarthritis (KOA) is a progressive multifactorial joint disease characterized by chronic pain, functional disability [1] and an increased incidence of comorbid psychological conditions [2]. Consequently, this condition represents one of the leading causes of physical impairment and global health burden [3].

Pain associated with KOA is recognized as a heterogeneous and complex phenomenon encompassing several mechanisms [4]. Although pain is traditionally associated with joint damage, there is evidence that a subgroup of this population does not show a direct association between severity of damage with pain intensity and level of disability [5,6]. For example, 19–43% of adults over 40 years with asymptomatic uninjured knees have features associated with osteoarthritis on MRI [5]. This suggests that osteoarthritis pain may not only be related to mechanisms involving cartilage degeneration but also to alterations in central pain processing. In this scenario, pain sensitization and altered pain modulation have been proposed as possible explanations for these cases [7]. Pain sensitization in KOA is often characterized by generalized hyperalgesia, impaired descending control of pain, and increased facilitation of temporal summation [7,8,9,10]. To achieve better outcomes, these possible altered pain mechanisms should be taken into consideration in the evaluation and treatment of patients with KOA. Psychophysical tests of somatosensory function, such as the pressure pain threshold (PPT), conditioned pain modulation (CPM) and temporal pain summation (TSP), are used to assess pain sensitization in patients with KOA [10]. Previous studies have shown that KOA patients exhibit impairment in all of them [11,12,13,14]. As well as it has been associated with disability and psychological distress [15].

At present, the highest quality guidelines consistently recommend education and exercise for the non-pharmacological treatment of pain and dysfunction caused by KOA [16]. However, exercise has only shown moderate effects for symptom and function improvement [17], and this is possibly because not all patients respond well to exercise [18,19]. This is why other strategies for patients with KOA to enhance the benefits of exercise-based physical therapy are needed [18].

Aligned with the above, transcutaneous electrical stimulation (TENS) has been shown to be useful in reducing pain and improving functionality in KOA patients [20]. The introduction of this type of current in a deeper manner through needles is generally referred to as percutaneous electrical nerve stimulation (PENS) and it has been proposed as a promising technique due to its observed effects [21,22,23]. Conceptually, both PENS and TENS use the same basic neurophysiological mechanisms. The “deep” versus “superficial” distinction reflects where and how strongly those mechanisms are engaged: PENS does this more focally and deeply by placing needles near nerves (for instance, observations in animal models have demonstrated an improvement in neuroinflammation) [24,25], while TENS does it more diffusely and superficially through the skin [26]. It has been hypothesized that PENS could generate a greater improvement than TENS, as the needle induces nociceptive activity, which in turn may trigger the activation of descending inhibitory and facilitatory pathways from the brain [27,28,29], and the addition of electricity to the needle would produce greater analgesia than the needle technique alone [30,31,32,33]. In addition, including PENS could improve the effect on pain sensitization, since, in isolation, it has been shown to produce effects in patients with musculoskeletal pain on PPTs and CPM [34] and this would allow control of the flare-ups so common in KOA [35], thus facilitating adherence to the exercise program. When this technique has been included in an exercise and manual therapy program for KOA, significant changes have been observed in the reduction in pain and increase in functionality, as well as a decrease in the intake of medications, with respect to the group that did not receive this intervention [36]. However, there is a need to improve knowledge of whether PENS has any significant effect on pain sensitization and psychological factors. Within a view of combined treatments, the rationale for applying pain education in conjunction with knee exercises has recently been presented [37]. It is hypothesized that the addition of PENS to a pain education and exercise program will produce a synergistic effect superior to both TENS and sham PENS, due to its potential impact on pain sensitization mechanisms. Therefore, the objectives of this pilot study are: (1) to explore whether adding PENS to a pain education and exercise program yields greater improvements in pain sensitization, physical function, psychological outcomes, and self-perceived improvement than adding a sham PENS intervention; (2) to explore whether any observed improvements are due to the application of deep electrical stimulation, or simply to the use of electrical stimulation with needle insertion, by comparing PENS with a control using TENS; and (3) to assess the feasibility, safety, and methodological integrity of the intervention, including participant recruitment and retention, adverse events, and the success of participant blinding. The estimation of the consistency and direction of these preliminary effects, together with feasibility and blinding outcomes, will guide the design and methodological optimization of future definitive clinical trials.

## 2. Materials and Methods

### 2.1. Study Design/Setting

A randomized, parallel group, assessor-blinded, sham-controlled pilot was undertaken at the Rey Juan Carlos University, Madrid, from July 2023 to February 2024. This study was approved by the Clinical Intervention Ethics Committee of Rey Juan Carlos University (Approval Number: 0801201900219) and registered in ClinicalTrials.gov Identifier: NCT05955430. Given the pilot nature of the study, no formal sample size calculation was performed. The sample size was determined pragmatically to assess feasibility outcomes and obtain preliminary estimates to inform future trials. All participants gave written informed consent before data collection began. Participants were informed that they would receive 1 of 3 electrical stimulation modalities (percutaneous, transcutaneous and inserted needles, or sham percutaneous). Study assessments were conducted by two independent and blinded investigators (L.R.-L. and B.S.G.), who were unaware of group assignments at all measurement points: baseline, post in-person intervention, post home-based intervention, and three-month follow-up. Two physiotherapists were responsible for delivering the interventions. One investigator (L.R.-L.), blinded to group allocation, administered the pain education and exercise program under blinded conditions. The different electrical stimulation interventions were applied by a separate investigator with over 10 years of experience, who was not blinded due to the impossibility of masking this part of the intervention. Participants were instructed to discontinue their use of analgesic pain medication before their first research appointment, as it could affect their pain perception thresholds and sensitivity to pain.

### 2.2. Participants

Thirty patients aged 45 years and older with painful KOA were recruited from the community in Madrid through local newsletters and social media (July–August 2023). The inclusion and exclusion criteria were based on a previous study in KOA with a special focus on cognitive change and physical activity [37]. See Supplementary Material for eligibility criteria (Table S1).

### 2.3. Outcomes Measures

Two physical therapists (L.R.-L. and B.S.G.), each with over five years of clinical experience and specific expertise in these types of evaluations, conducted the assessments at baseline, after the in-person intervention (1 month), following the home-based intervention (3 months), and at the three-month follow-up. Both were blinded to group allocation. Sociodemographic data such as age, sex, and body mass index were collected at baseline, before the first intervention. Outcome measures associated with pain severity and function were: Visual Analogue Scale (VAS), Chronic Pain Grading Scale (CPGS), WOMAC, The Timed Up and Go (TUG) Test and Short Physical Performance Battery (SPPB).

#### 2.3.1. Visual Analogue Scale

Pain intensity was measured with the VAS using a 10 cm line with endpoint descriptors such as ‘no pain’ marked at the left end of the line and ‘worst pain imaginable’ marked at the right end. Patients are asked to mark a point on the line that best represents their pain. The Minimal Clinically Important Difference (MCID) for VAS = −19.9 mm [38].

#### 2.3.2. Chronic Pain Grading Scale

Pain severity will be assessed using the Spanish version of the CPGS [39]. This scale is a self-reported instrument that consists of two subscales; the first assesses pain intensity, and the second assesses perceived disability. The scale is made up of a total of 8 items, 7 of which are 11 points in Likert format, and the other item assesses the perpetuation of pain, asking for the number of days of pain in the previous 6 months. The Spanish version of the CPGS has proven to be a valid and reliable instrument to assess the severity of chronic pain.

#### 2.3.3. Western Ontario and McMaster Universities Osteoarthritis Index Questionnaire

The functionality was measured with the Spanish version of WOMAC [40]. It contains 24 questions: 5 about pain (range: from 0 to 20 points), 2 about stiffness (range: from 0 to 8 points), and 17 about difficulty with physical functions (range: from 0 to 68 points) and can be completed in less than 5 min. An increase in the WOMAC scores indicates a greater degree of disability. The MCID was defined as the 75th percentile of the change in the function subscale score among patients with good response to treatment [38].

#### 2.3.4. The Timed up and Go Test

The TUG was used to assess mobility ability. The patient began seated in a chair with the back supported and arms on the armrests, upon hearing the word “Come on” they had to get up and walk to a mark on the floor 3 m away and finally the person had to turn around and walk back to sit in the chair. The time it took from saying “Let’s go” to sitting down again was timed (3 attempts and the results were averaged) [41].

#### 2.3.5. Short Physical Performance Battery

It allows classifying the level of physical performance of the elderly along the entire functional spectrum. It consists of three tests: balance (in three positions: feet together, semi-tandem and tandem), walking speed (4 m) and getting up and sitting in a chair five times. The average administration time is between 6 and 10 min. The total SPPB score is the sum of the three subtests and ranges from 0 (worst) to 12; changes of 1 point have clinical significance. A score below 10 indicates frailty and an elevated risk of disability as well as falls [42].

Outcome measures for pain sensitization were PPT, CPM and TSP.

#### 2.3.6. Pressure Pain Thresholds

The PPTs were evaluated by algometry (FDX-25 Wagner Instruments, Greenwich CT) with the subject seated. To record the UDP, a point was marked at the middle of the distal phalanx thumb, another at 1 cm medial to the patella (joint interline), another at the tibialis anterior (5 cm below the tibial tuberosity and 1 cm lateral to the tibial spine), and a last point at the middle of the belly of the upper trapezius (midpoint between acromion and C7). Each measurement was performed on the right or left side, depending on the most symptomatic side of the patient. Pressure was exerted with the applicator placed perpendicular to the skin and increased at a rate of 1 kg/s until the patient reported the onset of pain. Two measurements were taken at each point with an inter-measurement interval of 10 s, and the mean of the 2 measurements was considered PPT.

#### 2.3.7. Conditioned Pain Modulation

First, the test was explained to the patient. The test stimulus was measured by algometry in the middle of the distal phalanx of the thumb on the symptomatic side. Then, an occlusion cuff was placed around the arm contralateral to the PPT assessment and above the ulnar fossa. The subject was asked to raise his arm overhead, clench, and relax the hand for 1 min to decrease the amount of blood in the extremity. The arm was lowered, and the cuff was inflated to 240 mmHg/s. Each subject was asked to continue opening and closing the hand until a pain of 6/10 was felt (assessed by numerical scale where 0: no pain; 10: worst possible pain). If the pain exceeded 6/10, the pressure was reduced until the subject expressed a pain of 6/10. A PPT measurement was performed in parallel on the middle of the distal phalanx of the thumb on the symptomatic side. After this, the cuff pressure was released and after 1 min rest PPT was again assessed at the same point to assess CPM function [43,44].

#### 2.3.8. Temporal Summation of Pain

An algometer was used to induce TSP. The application surface was 1 cm^2^. It was applied vertically, directly on the distal phalanx of the thumb on the asymptomatic side (contralateral to the PPT assessment), stimulating 10 consecutive times for 1 s and with intervals of 1 s with a previously determined pressure on the PPT. Patients were asked to indicate on a numerical scale (0 to 10) the pain they felt in the first and last stimulus. The temporal summation was obtained from the difference between the first and tenth measurement of pain intensity [45].

Psychosocial factors involved in pain were assessed as they have been observed to contribute to the pain and functional deficits associated with osteoarthritis [46]: The State-Trait Anxiety Inventory (STAI), Beck Depression Inventory-II (BDI-II), Pain Catastrophizing Scale (PCS), Tampa Scale for Kinesiophobia (TSK-11). A full description of all secondary outcome measures is provided in the Supplementary Material (File S1).

### 2.4. Feasibility and Safety

Feasibility was assessed by recording participant retention throughout the study. Safety was monitored by documenting any adverse events reported during or after the intervention sessions. An adverse event was defined as any unfavorable or unintended sign, symptom, or disease temporally associated with the intervention, regardless of its relationship to the intervention.

### 2.5. Procedures and Intervention

**Figure 1:**
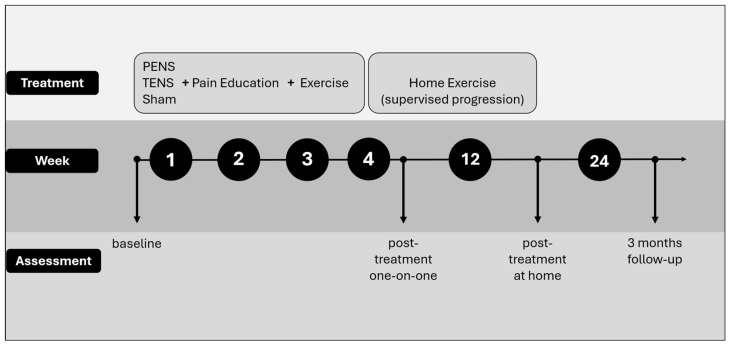
Summary of the assessments and interventions.

A fourth physical therapist (A.A.-R.) was responsible for the randomization of patients and for keeping the assignment concealed in one of the three groups: (a) PENS + Pain education and Exercise, (b) Control TENS + Pain education and Exercise, or (c) Sham PENS + Pain education and Exercise. For the randomization, a list of random numbers associated with patient codes was generated, and based on these, each patient’s assignment was placed in envelopes that only the stimulation interventionist had access to just before the intervention. A summary of the assessments and interventions is provided in Figure 1.

OARSI recommendations suggest, among other things, that therapeutic exercise be delivered on an individualized basis and accompanied by education [47]. In this study, the pain neuroscience education and individualized physical activity programs was developed using the core principles of the EPIPHA-KNEE protocol as a reference [37]. Their integrated approach was adapted to our clinical context without reproducing original materials.

#### 2.5.1. Therapeutic Exercise

It consisted of strengthening exercises and gradual walking. Lower extremity strengthening exercises were individualized and progressive for each patient for twelve weeks. Eight one-on-one sessions were conducted over four weeks. And then the patient continued those exercises for an additional eight weeks at home. In addition, they had to complete twelve weeks of progressive walking until they achieved at least 30 min, 5 days a week at moderate intensity (defined as moderate respiratory distress while walking). Home treatment adherence was recorded by means of a training diary. See detailed intervention and exercise instructions in Supplementary Material (Files S2 and S3 and Table S2).

#### 2.5.2. Pain Education

Participants received four 30 min one-on-one sessions (one session per week) of pain science education. The objective was to change the participants’ conceptualization of pain: from seeing pain as a marker of tissue damage to a marker of the body’s perceived need for protection. Contents included basic structure of the nervous system, distinction between nociception and pain, peripheral and central sensitization, neuroplasticity, and benefits of exercise. In addition, participants were given relevant multimedia content to watch at home, which was reviewed in the following session to check their understanding. The therapist avoided conflicting or contradictory messages regarding electrical stimulation. See detailed pain education program in Supplementary Material (File S4).

#### 2.5.3. PENS

A TENS current (100 Hz 100 µs) was applied through four needles located in the knee for 30 min. In case of bilateral pain, the most painful knee was chosen. Eight sessions were carried out for one month (two per week). The punctures were performed at the most painful point in each of the four zones [48]. The distribution of the channels was as follows: for the first channel, anode in lateral anterior zone and cathode in medial anterior zone, and for the second channel, anode in Hoffa’s fat and cathode in goose foot. Participants were able to adjust the amplitude to a strong and manageable intensity of sensory stimulation that was not strong enough to elicit muscle contraction. The equipment used was EPTE BIPOLAR SYSTEM V02^®^. See detailed intervention in Supplementary Material (File S5, Figures S1 and S2).

#### 2.5.4. Control TENS

A TENS current (100 Hz 100 µs) was applied through four patches placed on the knee for 30 min. Eight sessions were performed for one month (two per week). The distribution of the channels was the same as for PENS. Needles were also introduced in this group, to determine that the differences with the PENS group are due to the deep current and not the needle insertion. Participants were able to adjust the amplitude to a strong and manageable sensory stimulation intensity that was not strong enough to elicit muscle contraction.

#### 2.5.5. Sham PENS

To perform the puncture simulation, the needles were cut and sanded so that they were blunt and could not penetrate the skin. In this way, the patient felt a prick as if the needle had been inserted, but it was withdrawn. To simulate electrostimulation, the protocol established by Rakel et al. [49] was implemented, which consists of applying TENS at 100 Hz with a 100-µs pulse duration for 30 s, followed by a reduction in intensity for the next 15 s. Eight sessions were held over a month (two per week). For better blinding of the interventions, a sheet was placed vertically at hip height so that they could not see what was being applied to their knee.

### 2.6. Statistical Analysis

Baseline characteristics of each group were summarized using descriptive statistics: mean and standard deviation for continuous variables, and absolute and relative frequencies for categorical variables. Between-group comparisons at baseline were performed using one-way analysis of variance (ANOVA) for normally distributed continuous variables, and the Kruskal–Wallis test when normality was not met. For categorical variables, the Chi-square test was applied.

To analyze between-group differences and changes over time, linear mixed-effects models with random intercepts for participants were employed, accounting for the repeated-measures structure and within-subject correlations. Fixed effects included group (PENS, TENS control, sham PENS), time point (baseline, post-treatment one to one, post-treatment at-home, 3-month follow-up), and the group-by-time interaction. Wald chi-square statistics derived from the linear mixed-effects models were used to test the significance of fixed effects.

Model assumptions were assessed by visual inspection of Q-Q plots and the Shapiro–Wilk test. When residuals deviated from normality, appropriate transformations (square root or natural logarithm) were applied to the dependent variable, selecting the transformation that best approximated a normal distribution of residuals. Robust standard errors were used to ensure valid inference in transformed models. Marginal estimates were back-transformed to the original scale using the inverse function to facilitate clinical interpretation.

Post hoc comparisons between groups were conducted at each post-baseline time point (post-treatment one to one, post-treatment at-home, and 3-month follow-up), adjusting for the corresponding baseline value. *p*-values were reported after Bonferroni correction. Statistical significance was set at *p* < 0.05 for all tests.

Blinding success was evaluated at the end of the intervention by asking participants which treatment they believed they had received (“PENS”, “Placebo”, “TENS”, or “Don’t know”). The degree of blinding was quantified using Bang’s Blinding Index (BI) for each treatment arm and the James’ BI for the overall trial. “Don’t know” responses were considered incorrect guesses in the main analysis, as they reflect the participants’ inability to identify their assigned treatment, indicating successful blinding. A sensitivity analysis was also performed excluding these responses.

Statistical analyses were conducted in STATA (IC 16.1, StataCorp LLC, Lakeway Drive, College Station, TX, USA).

## 3. Results

**Figure 2:**
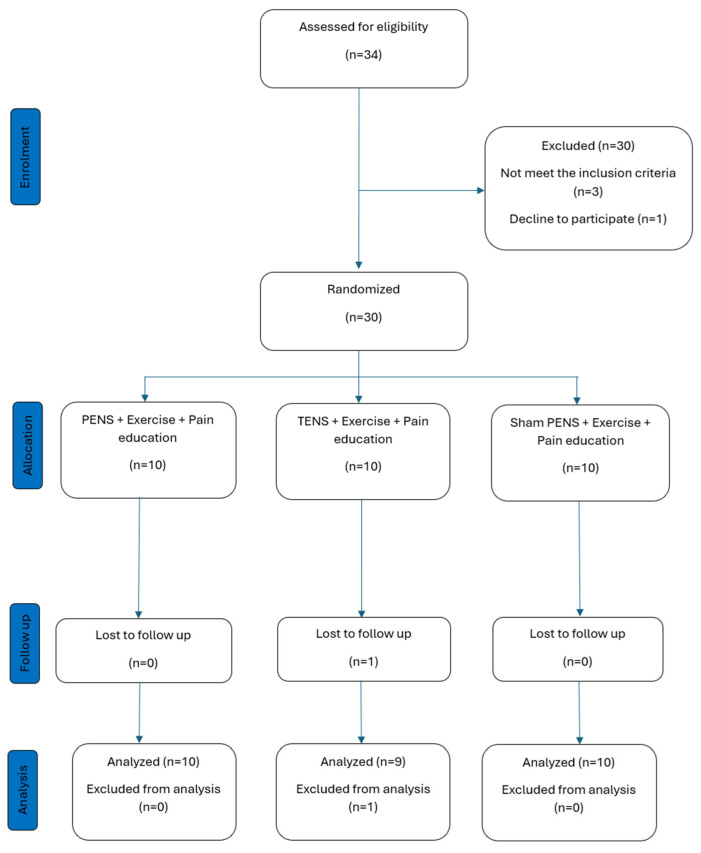
Process of recruitment and dropouts.

Thirty-four patients with KOA were screened for possible eligibility criteria. Of these 34, 3 were ineligible and 1 declined to participate. Thirty patients finally were randomized (9 males, 21 females); 10 patients in the PENS + pain education and exercise group (2 males, 8 females; mean age ± SD, 68.39 ± 10.24 years), 10 patients in the control TENS + pain education and exercise group (3 males, 7 females; mean age ± SD, 66.81 ± 7.85 years), and 10 patients in the sham stimulation + pain education and exercise group (4 males, 6 females; mean age ± SD, 66.00 ± 6.19 years). One participant in the TENS group withdrew from the study before the post-treatment assessment due to family complications and was therefore excluded from the analysis. Figure 2 shows the process of recruitment and dropouts. There were no significant differences between groups in terms of demographic or clinical characteristics at the time of the baseline screening. See baseline participant demographics and clinical measures in Table 1.

### 3.1. Feasibility, Adherence and Safety

Feasibility outcomes were favorable. Retention was high, with only one dropout (3.3%), and adherence to the exercise program was good: 82.8% of participants reported completing the prescribed home exercises according to the training diaries. No adverse events, such as dizziness, nausea, or increased pain were reported.

### 3.2. Effects on Pain Intensity and Function

**Figure 3:**
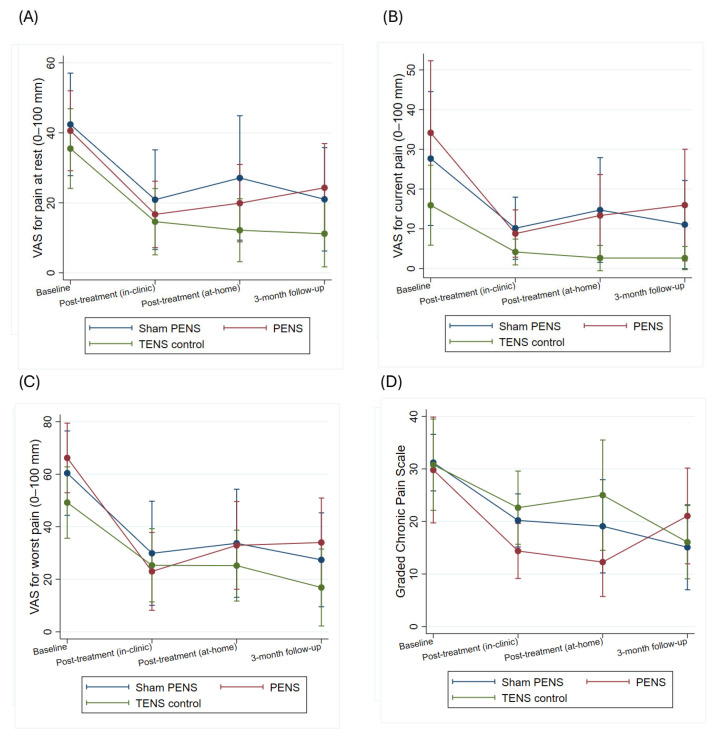
Effects of the interventions on pain outcomes: (**A**) VAS at rest, (**B**) current VAS, (**C**) worst VAS, and (**D**) CPGS. *Y*-axis scaled to observed data range to improve visual interpretation. PENS: Percutaneous electrical nerve stimulation; TENS: Transcutaneous electrical nerve stimulation; VAS: Visual analogue scale; CPGS: Chronic Pain Grading Scale.

**Figure 4:**
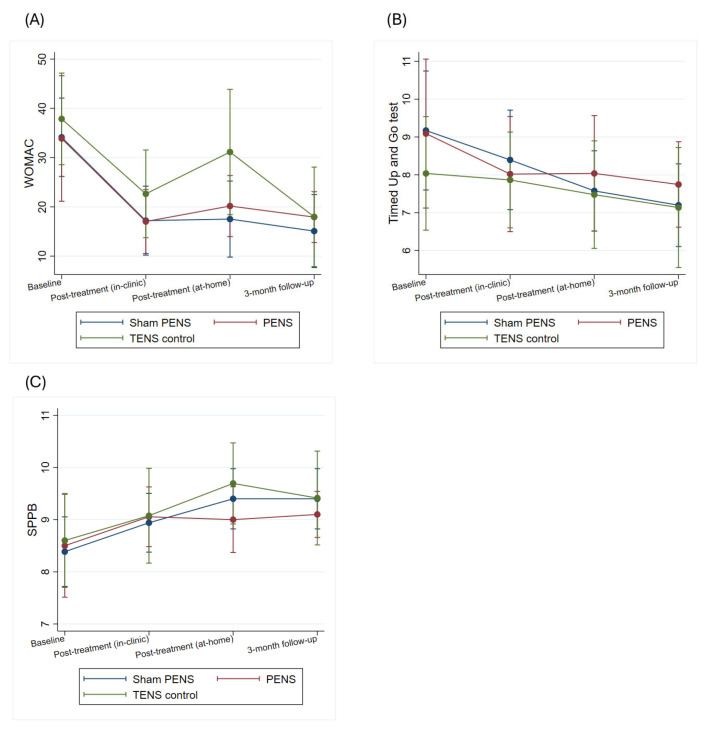
Effects of the interventions on physical function and disability: (**A**) WOMAC, (**B**) TUG, and (**C**) SPPB. *Y*-axis scaled to observed data range to improve visual interpretation. WOMAC: Western Ontario and McMaster Universities Osteoarthritis Index questionnaire; TUG: Timed Up and Go; SPPB: Short Physical Performance Battery.

The results show that in the variables related to pain intensity (VAS mean, VAS worst, CPGS) and in those related to function (WOMAC, TUG, SPPB) there was a significant effect of the time factor (VAS mean (Chi^2^ = 13.38; *p* = 0.004), VAS worst (Chi^2^ = 28,31; *p* < 0.001), CPGS (Chi^2^ = 15.22; *p* = 0.002), WOMAC (Chi^2^ = 31.44; *p* < 0.001), TUG (Chi^2^ = 23.30; *p* < 0.001), SPPB (Chi^2^ = 8.81; *p* = 0.012)), but not of the time-group interaction (VAS mean (Chi^2^ = 3.62; *p* = 0.727), VAS worst (Chi^2^ = 6.89; *p* = 0.331), CPGS (Chi^2^ = 11.33; *p* = 0.079), WOMAC (Chi^2^ = 4.61; *p* = 0.595), TUG (Chi^2^ = 8.80; *p* = 0.185), SPPB (Chi^2^ = 8.61; *p* = 0.197)). In Bonferroni adjusted post hoc comparisons there were no significant effects between any of the groups at any time point. In the case of pain intensity (VAS mean and VAS worst) and disability (WOMAC), the magnitude of within-group change observed across follow-up assessments exceeded commonly accepted minimal clinically important difference thresholds in most intervention arms and time points, suggesting clinically meaningful improvement over time. Pain intensities and disability scores obtained by patients are summarized in Figure 3 and Figure 4 and Supplementary Material (Table S3).

### 3.3. Effects on Pain Sensitization

**Figure 5:**
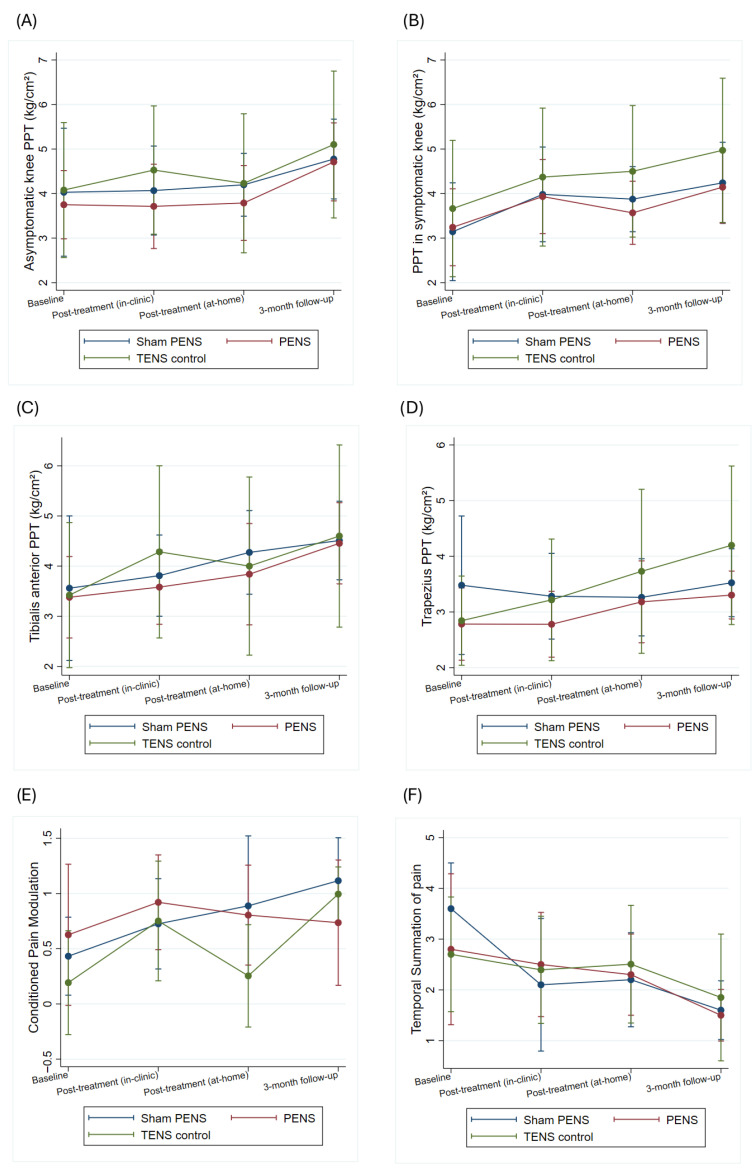
Effects of the interventions on pain sensitization measures: (**A**) PPT asymptomatic knee, (**B**) PPT symptomatic knee, (**C**) PPT tibialis anterior, (**D**) PPT trapezius, (**E**) CPM, and (**F**) TSP. *Y*-axis scaled to observed data range to improve visual interpretation. CPM: Conditioned pain modulation; PPT: Pressure pain thresholds; TSP: Temporal summation of pain.

A significant effect of the time factor was observed for TSP and CPM (CPM (Chi^2^ = 8.77; *p* = 0.032), TSP (Chi^2^ = 53.11; *p* < 0.001)), but not PPT, either local or remote local PPT (Chi^2^ = 6.21; *p* = 0.102), remote PPT (Chi^2^ = 2.64; *p* = 0.450). No significant group-by-time interaction was found for any of the psychophysical measures (local PPT (Chi^2^ = 1.84; *p* = 0.934), remote PPT (Chi^2^ = 7.21; *p* = 0.302), CPM (Chi^2^ = 5.97; *p* = 0.426), TSP (Chi^2^ = 5.23; *p* = 0.514)). Bonferroni-adjusted post hoc comparisons revealed no significant differences between any of the intervention groups at any time point. A summary of the psychophysical test results is presented in Figure 5 and Supplementary Material (Table S4).

### 3.4. Effects on Psychological Factors

**Figure 6:**
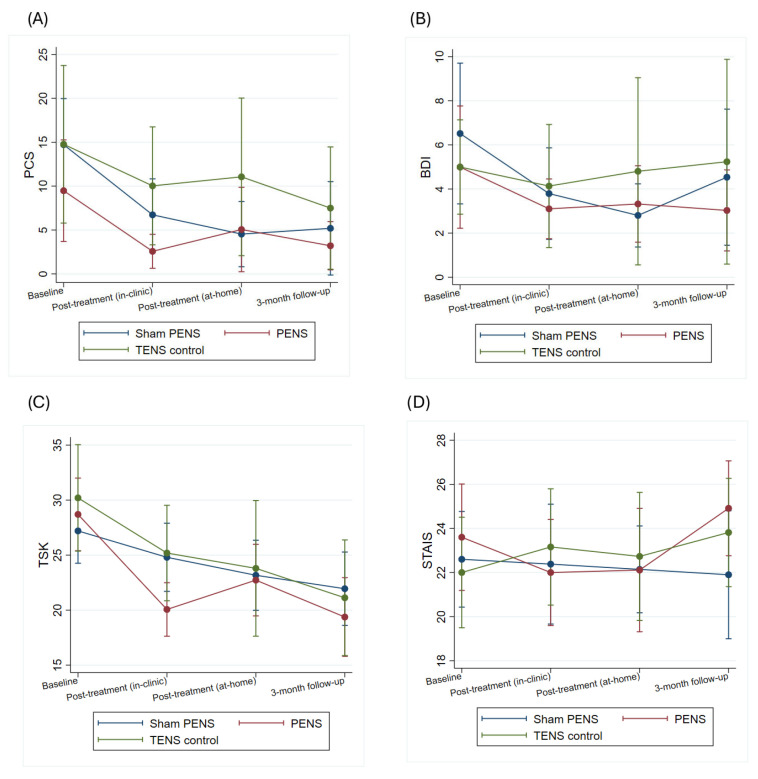
Effects of the interventions on psychological outcomes: (**A**) PCS, (**B**) BDI-II, (**C**) TSK, and (**D**) STAI. *Y*-axis scaled to observed data range to improve visual interpretation. PCS: Pain Catastrophizing Scale; BDI-II: Beck Depression Inventory-II; TSK Tampa Scale for Kinesiophobia; STAI: State-Trait Anxiety Inventory.

The results show a significant effect of the time factor for catastrophizing and depression (PCS (Chi^2^ = 41.48; *p* < 0.001), BDI-II (Chi^2^ = 11.23; *p* = 0.010)). In contrast, no statistically significant overall main effect of time was detected for kinesiophobia or anxiety (TSK (Chi^2^ = 5.45; *p* = 0.141), STAI (Chi^2^ = 0.29; *p* = 0.961)). No significant group-by-time interaction effects were found for any variable (PCS (Chi^2^ = 6.61; *p* = 0.359), BDI-II (Chi^2^ = 5.80; *p* = 0.445), TSK (Chi^2^ = 9.40; *p* = 0.152), STAI (Chi^2^ = 5.42; *p* = 0.491)). Bonferroni-adjusted post hoc comparisons revealed no significant differences between groups at any time point. The results of the psychological tests obtained by the patients are summarized in Figure 6 and Supplementary Material (Table S5).

### 3.5. Success of Blinding

Half of the participants (50%) responded “don’t know” when asked which treatment they believed they had received, indicating a generally good level of uncertainty. When “don’t know” responses were considered incorrect, the proportion of correct guesses was 50% in the PENS group, 10% in the sham PENS group, and 30% in the TENS group. The corresponding Bang’s Blinding Index (BI) values were 0.25, –0.35, and –0.05, respectively, suggesting effective blinding in the sham PENS and TENS groups and only a mild tendency toward unblinding in the PENS group. The overall James’ BI was 0.60, consistent with an acceptable level of blinding across the trial. A sensitivity analysis excluding “don’t know” responses yielded higher BI values (0.75 for PENS, 0.25 for sham PENS, and 0.14 for TENS), as expected when considering only participants who provided a specific guess.

## 4. Discussion

This pilot randomized controlled trial evaluated whether adding PENS to a pain education and exercise program would enhance its effects on pain severity and sensitization, physical function, psychosocial factors, and self-perceived improvement in patients with KOA. The underlying rationale was that the specific therapeutic effects of deep percutaneous electrical stimulation, over and above placebo and contextual influences, might reduce pain and pain sensitization between sessions, thereby facilitating greater engagement in exercise and leading to superior medium-term outcomes. To address this, PENS was compared with a sham PENS procedure, controlling for needle insertion and device-related contextual effects, and with a control TENS intervention in which electrical current was applied superficially. The latter was specifically designed so that, when contrasted with PENS, any differential effects could be attributed to the depth of current application rather than to the mere use of electrical stimulation or needle insertion. However, no significant between-group differences were observed for any clinical or mechanistic outcome, indicating that, under the conditions of this study, PENS did not demonstrate a clear advantage over sham PENS or superficial TENS when all groups concurrently received the same pain education and exercise program. Nevertheless, the intervention was feasible and well tolerated, with high retention and adherence rates, no relevant adverse events, and generally successful participant blinding, supporting the methodological integrity and safety of the study procedures.

All three intervention arms showed statistically and clinically meaningful improvements over time in pain intensity, CPGS, disability, physical function, CPM, TSP, pain catastrophizing, and depressive symptoms. These findings indicate that the overall intervention context, a combination of pain education, structured exercise, and the delivery of an apparently sophisticated technical procedure was effective in reducing symptom burden and, at least partially, in modifying pain processing and related psychosocial factors. In contrast, no significant time effects were observed for local or remote PPTs, anxiety, or kinesiophobia, suggesting that not all dimensions of sensitization or psychological distress were equally responsive within the time frame and dosage of the present protocol. Because no group received education and exercise alone, the respective contributions of the common program and the contextual or placebo effects associated with PENS, TENS, and sham PENS cannot be disentangled. Moreover, the magnitude of within-group improvements in several key outcomes across all three arms raises the possibility of a ceiling effect, whereby patients derive substantial benefit from the multimodal conservative approach and its contextual components, leaving limited scope for additional gains specifically attributable to deep percutaneous electrical stimulation.

The influence of contextual and placebo mechanisms is especially relevant when interpreting these findings. All three interventions involved needle procedures and the use of an electrical device, elements that are known to carry a strong therapeutic ritual and to shape patients’ expectations and meaning responses. As highlighted by Rossettini et al. [50], contextual factors such as clinician–patient interaction, treatment credibility, invasiveness, and the perceived technological sophistication of an intervention can substantially modulate clinical outcomes in musculoskeletal pain, sometimes approaching the magnitude of specific treatment effects. In the present trial, PENS, control TENS, and sham PENS were all embedded in a rich contextual framework and delivered alongside a potent common intervention of education and exercise. Under these conditions, it is plausible that contextual and placebo-related effects contributed meaningfully to the improvements observed in all groups, and that any specific physiological advantages of deep percutaneous electrical stimulation were diluted or rendered undetectable against this strong shared background. This consideration is particularly relevant given the absence of an education and exercise only control group, which limits the separation of specific, common, and contextual treatment effects.

From a mechanistic standpoint, these results should also be interpreted within the complex and heterogeneous nature of pain in KOA. Pain in this condition is influenced not only by structural joint changes but also by peripheral and central sensitization [51], psychosocial factors, metabolic status, and proposed osteoarthritis phenotypes (e.g., inflammatory, metabolic, or mechanical overload profiles). It is therefore unlikely that a single, uniformly applied intervention such as PENS will exert the same effect across all patients. In addition, PENS and TENS have been shown to induce short-term reductions in pain and pain sensitization [34], which suggests that their greatest clinical utility may lie in providing transient analgesia rather than in producing large medium-term changes when delivered according to a fixed schedule. In this regard, it is conceivable that these techniques might be more effective if implemented in a symptom-contingent manner, targeting days of increased pain or flare-ups that limit exercise participation, rather than as protocolized sessions on predetermined days. Future studies should explore whether adapting the timing of electrostimulation to periods of heightened pain could better support engagement in exercise and yield more pronounced benefits than the fixed dosing regimen used in the present trial. Alternatively, the absence of between group differences may reflect the dose response characteristics of electrostimulation, as the number of sessions delivered, while consistent with clinical practice, may not have been sufficient to generate effects beyond those achieved by the concurrent education and exercise program at either short- or medium-term follow up.

Symptom fluctuation and episodic flares are highly characteristic of KOA [52,53] and movement-evoked pain is a major barrier to activity-based interventions [54]. At present, there is no solid evidence to support or refute any specific non-pharmacological therapy for the clinical management of flares in this population [55], and the present pilot data do not allow PENS to be recommended as an effective tool for this indication. This underscores the need to identify safe, non-pharmacological strategies that can address flare-ups while maintaining adherence to exercise. Recent evidence suggests that manual therapy may offer a safe option for short-term pain relief in patients with KOA [56], illustrating the potential value of time-limited, symptom-targeted interventions. Within this evolving landscape, the specific role of PENS in the management of flares remains uncertain and warrants further investigation in adequately powered, symptom-contingent trial designs.

Baseline psychological scores in this sample were generally low, particularly for anxiety and depressive symptoms, and low to low–moderate for kinesiophobia and pain catastrophizing. Under these conditions, the magnitude of change achievable over time is inherently limited, and any improvements may be small, potentially resulting in non-significant findings or changes in limited clinical relevance. It is therefore plausible that patient subgroups or phenotypes characterized by greater psychological comorbidity might show more clearly detectable benefits from this type of multimodal intervention, especially given that both exercise and pain education have demonstrated favorable effects on psychological variables in chronic pain populations [16]. Future adequately powered studies should further explore the relationships between psychological factors and pain related outcomes, as well as their potential role in moderating treatment response.

Future research should aim to identify patient profiles characterized by clear pain sensitization and pain limited exercise capacity. In such subgroups and considering the known short-term effects of PENS on pain processing, PENS may function as an adjunct to facilitate exercise participation and adherence, potentially translating into greater functional gains.

### 4.1. Clinical Relevance

From a clinical perspective, these findings primarily highlight the value of a multimodal conservative approach based on pain education and exercise for patients with KOA. All three groups, including those receiving sham PENS and superficial TENS, showed clinically meaningful improvements in pain, disability, and several mechanistic and psychosocial outcomes, underscoring the relevance of this type of intervention in routine practice. These results are consistent with evidence indicating that exercise therapy not only improves symptoms and impairments in people with KOA but also exerts broader positive effects on overall health and well-being [57,58], and with data supporting the role of pain education in addressing psychological comorbidities that contribute to pain persistence in chronic pain populations [59]. Within this framework, the present pilot data does not suggest that PENS provides benefits clearly exceeding those associated with sham PENS or control TENS; nevertheless, they do not exclude the possibility that PENS exerts specific short-term effects on pain processing. Indeed, pain in KOA is thought to involve maladaptive changes in both peripheral structures and the central nervous system, contributing to hyperalgesia and impaired descending pain inhibition [60]. Although these effects appear to be transient, they may offer clinically meaningful symptom relief for selected patients. For example, when surgery is contraindicated or when other conservative options prove insufficient and TENS can be safely self-administered at home. Considering the present results, education, exercise, and weight management should remain the core first-line strategies, while needling-based electrostimulation techniques may be considered as adjunctive tools within individualized, multimodal pain management rather than as routine enhancers of such programs.

### 4.2. Strengths and Weaknesses

The main strength of this study is that it evaluates the feasibility and potential added value of PENS when integrated into a guideline-based program of pain education and exercise in KOA, a setting in which recommended conservative treatments do not achieve satisfactory outcomes in all patients. The three-arm randomized, sham-controlled design (PENS, control TENS and sham PENS), embedded within the same multimodal framework, offers preliminary and clinically relevant information on how different electrostimulation strategies may interact with best-practice conservative care. The study also provides useful data on feasibility, acceptability, safety and blinding, which are crucial to inform the design of larger confirmatory trials.

However, the pilot nature and limited sample size restrict statistical power and preclude firm conclusions regarding comparative efficacy or the detection of clinically meaningful between group differences. Accordingly, the absence of statistically significant inter-group effects should be interpreted as exploratory rather than confirmatory. Moreover, the absence of a group receiving pain education and exercise alone prevents disentangling the specific effects of electrostimulation from those of the common intervention and its associated contextual or placebo components. In addition, weight management was not targeted during the intervention, and all groups presented mean BMI values in the overweight range, which may have influenced individual responses given the links between excess body weight, inflammation and nociceptor sensitization [61].

The fixed dosing and scheduling of electrostimulation and the short follow-up period further limit the interpretation of mechanistic and long-term effects. These considerations underscore the need for larger, adequately powered trials, including education and exercise, only arm and explicit weight management strategies to better define the role of PENS as an adjunct to conservative treatment in KOA.

## 5. Conclusions

Within the context of the present trial, the addition of PENS to a pain education and exercise program was not associated with additional improvements in pain sensitization, physical function, or psychological factors compared with sham PENS or control TENS. Importantly, the intervention proved to be feasible, safe, and generally well blinded, supporting the methodological viability of future adequately powered trials to further explore the role of PENS, potentially targeting patient profiles with clear pain sensitization and pain limited exercise capacity, where PENS could serve as an adjunct to enhance exercise adherence and maximize functional gains.

## Figures and Tables

**Table 1:** Participant demographics and baseline outcomes.

	PENS (*n* = 10)	Control TENS (*n* = 10)	Sham PENS (*n* = 10)	Differences Between Groups	
				Statistic	*p* Value
**Sex**					
Female, *No. (%)*	8 (80)	7 (40)	6 (60)	Chi^2^ = 0.95	0.621
Male, *No. (%)*	2 (20)	3 (30)	4 (40)		
**Age (y)**, *mean (SD)*	68.39 (10.24)	66.81 (7.85)	66.00 (6.19)	F = 0.22	0.808
**Weight (kg)**, *mean (SD)*	77.30 (12.14)	73.30 (11.23)	76.60 (12.56)	F = 0.32	0.731
**Height (m)**, *mean (SD)*	1.64 (0.09)	1.65 (0.08)	1.65 (0.10)	F = 0.03	0.968
**BMI (kg/m^2^)**, *mean (SD)*	28.74 (3.81)	26.88 (3.35)	28.26 (3.95)	F = 0.68	0.5160
**Marital status**				Chi^2^ = 2.59	0.858
Single, *No. (%)*	0 (0)	1 (10)	0 (0)		
Married, *No. (%)*	7 (70)	7 (70)	8 (80)		
Separated or divorced	2 (20)	1 (10)	1 (10)		
Widowed, *No. (%)*	1 (10)	1 (10)	1 (10)		
**Cohabitation**				Chi^2^ = 1.88	0.759
Alone, *No. (%)*	1 (10)	2 (20)	2 (20)		
Pair, *No. (%)*	6 (60)	5 (50)	7 (70)		
Family, *No. (%)*	3 (30)	3 (30)	1 (10)		
**Support for daily living activities**				Chi^2^ = 2.07	0.355
No, *No. (%)*	10 (100)	10 (100)	9 (90)		
Yes, *No. (%)*	0 (0)	0 (0)	1 (10)		
**Level of education**				Chi^2^ = 12.60	0.126
Elementary school, *No. (%)*	7 (70)	5 (50)	3 (30)		
Secondary, *No. (%)*	0 (0)		2 (20)		
Bachelor’s degree or Professional training, *No. (%)*	3 (30)	0 (0)	4 (40)		
University, *No. (%)*	0 (0)	3 (30)	1 (10)		
**Take painkillers**				Chi^2^ = 2.07	0.355
No, *No. (%)*	9 (90)	10 (100)	10 (100)		
Yes, *No. (%)*	1 (10)	0 (0)	0 (0)		
**Previously treated with PENS**				Chi^2^ = 0.373	0.830
No, *No. (%)*	10 (100)	9 (90)	8 (80)		
Yes, *No. (%)*	0 (0)	1 (10)	2 (20)		
**Previously treated with TENS**				Chi^2^ = 2.22	0.329
No, *No. (%)*	8 (80)	2 (20)	8 (80)		
Yes, *No. (%)*	2 (20)	8 (80)	2 (20)		
**Pain duration (mo)**, *mean (SD)*	75.70 (92.05)	77.40 (48.37)	80.80 (82.71)	Chi^2^ = 0.70	0.703
**Previous sessions**, *mean (SD)*	4.40 (5.23)	5.60 (4.20)	4.80 (5.69)	Chi^2^ = 1.75	0.417
**Exercise (days per week)**, *mean (SD)*	4.30 (2.36)	3.30 (2.58)	2.80 (2.25)	F = 1.01	0.377
**Pain intensity (0–100 cm)**					
Current, *mean (SD)*	40.20 (27.03)	19.90 (16.22)	34.10 (26.22)	F = 1.94	0.164
Mean in the last week, *mean (SD)*	40.60 (19.10)	35.50 (19.05)	42.40 (24.50)	F = 0.29	0.751
Worst in the last week, *mean (SD)*	66.20 (22.13)	49.20 (22.71)	60.40 (26.85)	F = 1.30	0.290
**WOMAC,** *mean (SD)*	36.90 (21.57)	39.30 (14.50)	35.30 (12.79)	F = 0.14	0.866
**TUG (s),** *mean (SD)*	9.74 (4.54)	8.41 (2.79)	9.52 (2.75)	Chi^2^ = 2.27	0.321
**SPPB,** *mean (SD)*	8.50 (1.65)	8.60 (1.51)	8.25 (1.04)	Chi^2^ = 1.515	0.469
**PPT (kg/cm^2^)**					
Symptomatic Knee, *mean (SD)*	3.39 (1.39)	4.07 (3.21)	3.39 (1.71)	Chi^2^ = 0.37	0.832
Asymptomatic Knee, *mean (SD)*	3.85 (1.22)	4.43 (3.07)	4.35 (2.15)	Chi^2^ = 1.58	0.453
Tibialis anterior, *mean (SD)*	3.50 (1.40)	3.81 (2.85)	3.93 (2.51)	Chi^2^ = 1.55	0.46
Trapezius, *mean (SD)*	2.78 (1.09)	2.84 (1.34)	3.48 (2.08)	F = 0.61	0.550
**CPM,** *mean (SD)*	0.63 (1.07)	0.19 (0.79)	0.43 (0.59)	F = 0.67	0.519
**TSP,** *mean (SD)*	2.80 (2.49)	2.70 (1.89)	3.60 (1.51)	F = 0.61	0.552
**PCS (0–52),** *mean (SD)*	11.70 (10.57)	18.20 (14.88)	15.90 (7.65)	F = 0.83	0.446
**BDI-II (0–63),** *mean (SD)*	6.30 (5.01)	5.50 (4.38)	8.60 (6.40)	Chi^2^ = 0.31	0.857
**STAI (0–60),** *mean (SD)*	23.60 (4.03)	22.00 (4.19)	22.60 (3.63)	F = 0.42	0.663
**TSK (0–44),** *mean (SD)*	28.70 (5.52)	30.20 (8.09)	27.20 (4.92)	F = 0.56	0.577

BDI-II: Beck Depression Inventory; BMI: Body mass index; CPM: Conditioned pain modulation; m: meter; mo: months; PCS: Pain Catastrophizing Scale; PENS: Percutaneous electrical nerve stimulation; PPT: Pressure pain thresholds; s: seconds; SPPB: Short Physical Performance Battery; STAI: State Anxiety Inventory; TENS: Transcutaneous electrical nerve stimulation; TSK: Tampa Scale for Kinesiophobia; TSP: Temporal summation of pain; TUG: Timed Up and Go; WOMAC: Western Ontario and McMaster Universities Osteoarthritis Index questionnaire; y: years.

## Data Availability

The raw data supporting the conclusions of this article will be made available by the authors upon request.

## Supplementary Materials

The following supporting information can be downloaded at: https://www.mdpi.com/article/10.3390/jcm15020624/s1, Table S1. Eligibility criteria, File S1. Secondary outcome measures, File S2. Detailed exercise program, Table S2. Graded strengthening program, File S3. Exercise instructions for patients, File S4. Detailed pain education program, File S5. Detailed PENS intervention, Figure S1. Knee pain map used for needles application, Figure S2. PENS intervention, Table S3. Mixed-effects model results for pain and disability outcomes, Table S4. Mixed-effects model results for pain sensitization measures, Table S5. Mixed-effects model results for psychological outcomes.

## Abbreviations

The following abbreviations are used in this manuscript:

PENS	Percutaneous electrical nerve stimulation
KOA	Knee osteoarthritis
TENS	Transcutaneous electrical nerve stimulation
WOMAC	Western Ontario and McMaster Universities Osteoarthritis Index
VAS	Visual Analogue Scale
CPGS	Chronic Pain Grading Scale
PPT	Pressure Pain Threshold
CPM	Conditioned Pain Modulation
TSP	Temporal Summation of Pain
TUG	Timed Up and Go
SPPB	Short Physical Performance Battery
STAI	State-Trait Anxiety Inventory
BDI-II	Beck Depression Inventory-II
PCS	Pain Catastrophizing Scale
TSK-11	Tampa Scale for Kinesiophobia
