# Supplementary material for: Feasibility and Preliminary Effects of Adding Percutaneous Electrical Nerve Stimulation to a Pain Education and Exercise Program in Patients with Knee Osteoarthritis: A Pilot Randomized Controlled Trial
Source: J Clin Med. 2026 Jan 13;15(2):624. doi: 10.3390/jcm15020624 (PMC12841921; doi:10.3390/jcm15020624)
Supplement: Supplementary file 1 [file jcm-15-00624-s001.zip › jcm-4042608-supplementary.pdf]

Table S1. Eligibility criteria

Inclusion	Exclusion
• Age ≥ 45 years<br>• Have activity-related joint pain<br>• Have morning stiffness ≤30 min<br>• Knee pain of at least 6 months duration<br>• OA diagnosis by physician	• Knee surgery on the most painful knee at present<br>• Health conditions that prevent safe participation in physical activity interventions (e.g., heart or lung disease)<br>• Neurological disorders affecting lower limb movement (e.g., multiple sclerosis or stroke)<br>• Inflammatory arthritis (including rheumatoid arthritis)<br>• Fibromyalgia<br>• Score of ≤ 23 points on the Mini-Cognitive Examination (MEC)<br>• Currently undergoing regular, active intervention for the knee (e.g., seeing a physiotherapist)<br>• Unable to commit to study requirements (e.g., attend to study appointments)

File S1. Secondary outcome measures

Mini-Cognitive Examination (MEC)

The MEC is the Spanish version of the Mini-Mental State Examination, the most widely used cognitive screening test to assess suspected symptoms compatible with cognitive impairment or dementia. The MEC consists of 30 items divided into 11 sections where temporal and spatial orientation, fixation, attention and calculation, memory, nomination, repetition, comprehension, reading, writing and drawing are assessed. The accepted cutoff point is > 23, anything lower is interpreted as a cognitive deficit.

The State-Trait Anxiety Inventory (STAI)

This questionnaire comprises two subsections of 20 items each for the measurement of anxiety as a state and as a trait, with a 4-point Likert-type response (0: not at all; 3: very much). Scores range from 0 to 60 points, with higher scores indicating greater anxiety. Consider the cut-off score at >= to 41.

Beck Depression Inventory-II (BDI-II)

BDI-II is a self-report measure of depression in a variety of settings and populations. It is the most widely used questionnaire worldwide to assess depression. The total score ranges from 0 to 63 points. 0-13, minimal depression; 14-19, mild depression; 20-28, moderate depression; and 29-63, severe depression. A change of 5 points corresponds to a minimally important clinical difference.

Pain Catastrophizing Scale (PCS)

The Spanish version of the PCS it has 13 items and each one is rated on a 5-point scale: 0 (not at all) to 4 (all the time). It comprises 3 dimensions: rumination, magnification, and despair. The theoretical range of the instrument is between 13 and 62, with low scores indicating low catastrophism and high values indicating high catastrophism.

Tampa Scale for Kinesiophobia (TSK-11)

TSK-11 is an 11-item scale that assesses the degree of fear of movement and (re)injury. Each item is scored from 1 to 4 according to the degree of agreement with the statement (1: do not agree at all; 4: strongly agree). The validated Spanish version of the 11-item scale has a total score of 11 to 44 items and has two subscales: activity avoidance and harm. Consider the cut-off score at >= to 34.

File S2. Detailed exercise program

The goal is for participants to walk at least 30 minutes, 5 days a week at a moderate intensity (defined as moderate shortness of breath while walking). With gradual increases in duration, number of walks, intensity, different terrains, among other characteristics.

Week 1: The initial phase is dedicated to establishing a baseline for each participant. Over 3-4 walks, the individual's unique walking tolerance is determined. Tolerance is operationally defined as the point just before a significant increase in knee symptoms occurs, quantified as a 2-point increase on a Numerical Rating Scale (NRS) for pain, visually noticeable swelling, or symptoms that persist for approximately two hours post-walk. During this week, and through Week 4, the therapist provides in-person guidance to assist the participant in scheduling their walks.

Week 2: The program formally commences using the data gathered in Week 1. The therapist calculates the average time or distance from the baseline walks and sets the "start" walking level at 80% of this value. The frequency of walks is also increased by one additional day. Furthermore, this week involves facilitated, individualized goal setting, where the therapist assists the participant in establishing short- and long-term activity-related goals to guide the personalization of the subsequent program.

Week 3: The progression continues with a focus on duration. The participant increases their walking time or distance to 90% of their original baseline tolerance, while the total number of walks (4-5) remains constant from the previous week.

Week 4: In this final week of the initial progression phase, the participant advances to walking at 100% of their baseline tolerance level. The frequency of walks is maintained at 4-5 sessions for the week.

Weeks 5 to 12: This extended phase focuses on steady progression towards the program's ultimate activity goal. Progression is managed on a weekly basis by either increasing the walking duration by 10% or by adding an additional walking day, but not both simultaneously. The choice between increasing duration or frequency is individualized for each participant. From Week 5 to 8, support transitions to video call sessions, where the physiotherapist assists the participant in updating their program, with the aim of fostering the participant's independence in managing their own progression. A mid-phase goal review is conducted in Week 8. The program concludes with a telehealth session at Week 12, during which the therapist assists the participant in planning for subsequent weeks and conducts a final review of the short- and long-term goals.

Table S2. Graded strengthening program

The intervention consisted of a 12-week program of lower-limb strengthening exercises that were individualized for each participant and progressively increased in both intensity and difficulty.

Week	Phase / Contact	Key Exercises & Progression
**1**	Baseline	No exercises; establishment of baseline walking tolerance.
**2-4**	Introduction & Buildup	**Wall Squats:** Increase from 1x10 to 3x10, 2x/week. **Sliding/Stepping:** Introduced and maintained at 1x10, 2x/week. **Sit-to-Stand:** Introduced in Week 4 at 1x10, 2x/week.
**5-6**	Video Calls	**Sit-to-Stand & Stepping:** Volume increased from 2x10 to 3x10, 2x/week.
**7-9**	Video Calls → At-home	**Step-ups & Step-downs (low step):** Introduced in Week 7; volume increased from 1x10 to 3x10, 2x/week.
**10-12**	At-home	**Step-ups & Step-downs (Advanced):** Increased step height; maintained at 3x10, 2x/week.
**13**	Conclusion	Strengthening exercises are optional to continue.

Abbreviations: reps = repetitions; 2x/week = two sessions per week.

File S3. Exercise instructions for patients

1. Wall squats

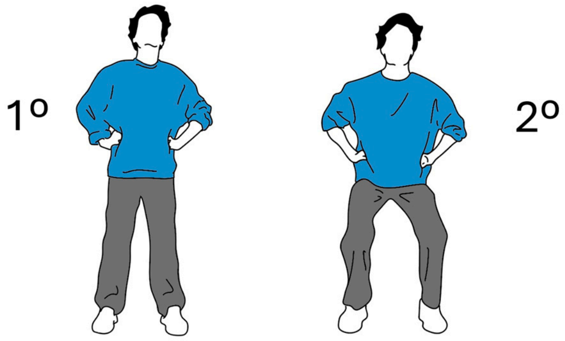

a) Lean against wall. Shoulders, back and buttocks resting against wall
b) Step feet 30cm from wall, hip-width apart
c) Slowly slide down and back up wall. Stop before knees cover toes
d) Hold 3-5 seconds at bottom of slide, slowly up

2. Sliding

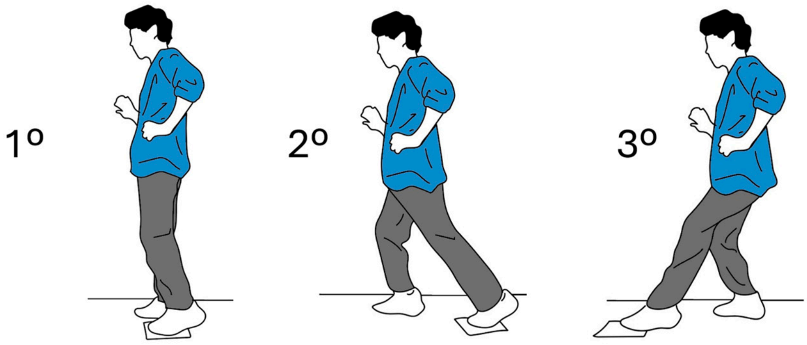

a) Standing on the sore leg
b) Painless leg on the sliding surface
c) Slowly slide forwards and backwards with painless leg
d) Start with smaller slides (few inches). If balance support needed, use wall

3. Sit-to-stand

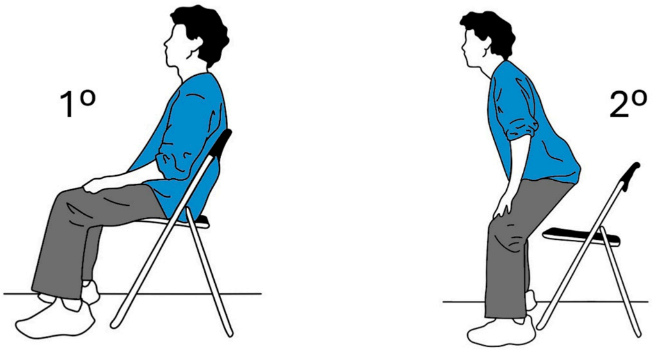

a) Sit on stable chair. Chair back against wall if support needed. Rest hands together on lap
b) Lean slightly forward. Move body weight forwards, nose over knees. Slowly up and slowly down

4. Stepping (forward/backwards)

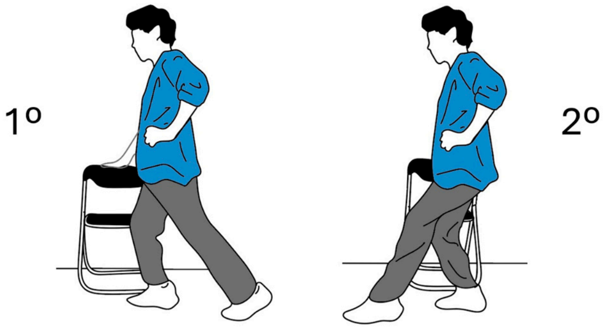

a) Stand on the sore leg. Wall (or back of chair) for balance support
b) Slowly step painless leg forward to touch floor in front
c) Step backwards again to starting position
d) Keep study leg slightly bent with weight on study leg throughout the exercise

5. Step Ups

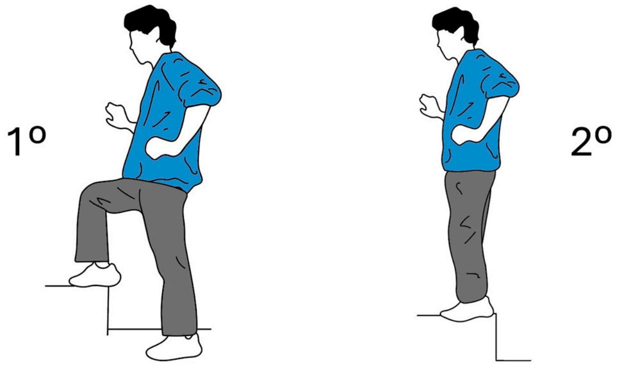

a) Stand with the sore leg on a step. Wall for balance support
b) Step up slowly taking body weight through sore leg

6. Step Downs

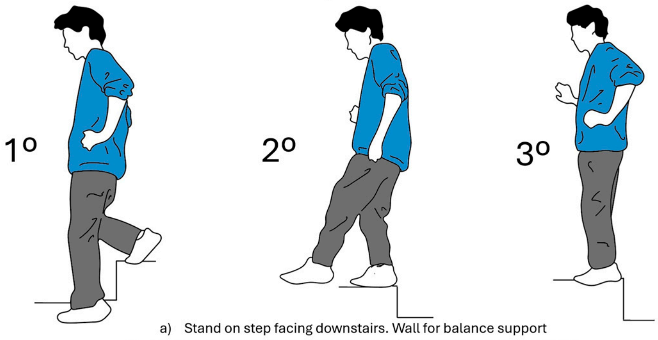

a) Stand on step facing downstairs. Wall for balance support
b) Bend the sore knee slowly and lower painless leg towards the ground
c) Keep the sore knee pointing forward during the movement
d) Straighten the sore knee slowly to return to starting position

File S4. Detailed pain education program

4 sessions of 30 minutes (1 session per week). Content:

This pain education program for KOA is designed to fundamentally reconceptualize the patient's understanding of pain. The primary aim is to shift the perception of pain from a direct marker of tissue damage to an indicator of the body's perceived need for protection, framing it as a protective feature of the nervous system. The program is structured around three key recovery ingredients: increasing awareness, increasing activity, and reducing inflammation. Educational content covers the basic function of the nervous system, distinguishing nociception from pain, and explaining concepts such as peripheral and central sensitization, which contribute to a "hyper-protective" state. A core principle taught is bioplasticity, emphasizing the adaptability of all bodily tissues and systems, including the pain system. The program actively counters misconceptions about activity, teaching that physical exercise does not increase joint damage but is beneficial, and introduces the "safe zone" for optimal activity dosing. Furthermore, it addresses the threat of radiological findings by discussing the poor correlation between X-ray results and pain, aiming to deconstruct associated fear and anxiety. Finally, the walking and strengthening components are framed as safe methods to rebuild physical capacity and, importantly, confidence in movement within a positive and recreational context.

File S5. Detailed PENS intervention

Group A: subjects assigned to this group underwent four weeks of PENS treatment. The punctures were performed in the four points that hurt most during walking in patients with knee pain of articular origin, described by Ikeuchi et al. 2013 (anterior medial, anterior lateral, goose foot and Hoffa's fat). Patient in supine position with semi-flexed knees. A total of four needles were used, making a single incision and then adapters were connected to apply TENS-type current to the needle. The distribution of the channels was as follows: for the first channel, anode in lateral anterior zone and cathode in medial anterior and for the second channel, anode in Hoffa's fat and cathode in goose foot. Subsequently, a TENS current (100 Hz 100 µs) was applied.

The application time of the current was 30 minutes. Participants could adjust the amplitude to a strong and manageable sensory stimulation intensity that was not strong enough to cause muscle contraction. The equipment used was EPTE BIPOLAR SYSTEM V02®.

PENS: 30 minutes 100 Hz at 4 points:

- perpendicular insertion within the vastus lateralis, three fingers proximal to the superolateral border of the patella.

- perpendicular insertion within the vastus medialis, three fingers proximal to the superomedial border of the patella.

- insertion perpendicular to the goose foot, two fingers medial to the tibial tuberosity.

- insertion medial to lateral within the infrapatellar fat, one finger distal to the inferior border of the patella.

Figure S1. Knee pain map used for needles application

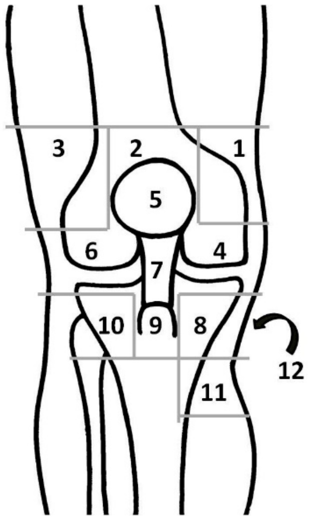

Figure S1. Knee pain map used for needles application (Ikeuchi et al. 2013).

Figure S2. PENS intervention

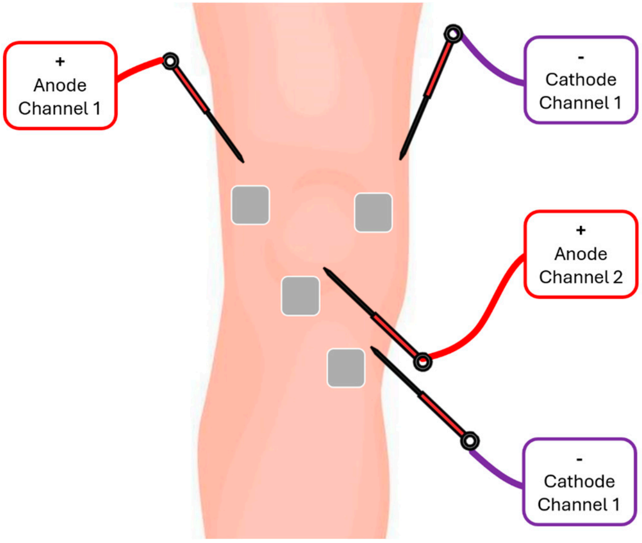

Figure S2. PENS intervention

Table S3. Mixed-effects model results for pain and disability outcomes

		**PENS** Margins (CI 95%)	**Control TENS** Margins (CI 95%)	**Sham PENS** Margins (CI 95%)	**Time factor** Chi² (p value)	**Group*Time factor** Chi² (p value)	**Pos-hoc comparisons** p value (Bonferroni)
**VAS mean**	Baseline	40.6 (29.18, 52.02)	35.5 (24.11, 46.89)	42.4 (27.75, 57.05)	13.38 (0.004)	3.62 (0.727)	
	Post-treat one-on-one	16.7 (7.21, 26.18)	14.61 (5.13, 24.08)	20.9 (6.67, 35.13)			PENS vs SHAM: 1.000 TENS vs. SHAM: 1.000 PENS vs. TENS: 1.000
	Post-treat at home	19.9 (8.84, 30.96)	12.16 (3.17, 21.15)	27.1 (9.32, 44.88)			PENS vs SHAM: 1.000 TENS vs. SHAM: 0.532 PENS vs. TENS: 1.000
	3 months follow-up	24.3 (11.67, 36.93)	11.16 (1.69, 20.63)	21 (6.21, 35.78)			PENS vs SHAM: 1.000 TENS vs. SHAM: 1.000 PENS vs. TENS: 0.575
**VAS worst**	Baseline	66.2 (52.96, 79.43)	49.2 (35.61, 62.78)	60.4 (44.34, 76.45)	28.31 (<0.001)	6.89 (0.331)	
	Post-treat one-on-one	23 (8.19, 37.81)	25.31 (11.37, 39.25)	29.9 (10.07, 49.72)			PENS vs SHAM: 0.893 TENS vs. SHAM: 1.000 PENS vs. TENS: 0.335
	Post-treat at home	32.9 (16.20, 49.60)	25.20 (11.71, 38.69)	33.7 (13.12, 54.27)			PENS vs SHAM: 1.000 TENS vs. SHAM: 1.000 PENS vs. TENS: 1.000
	3 months follow-up	34 (17.07, 50.92)	16.87 (2.23, 31.50)	27.4 (9.52, 45.27)			PENS vs SHAM: 1.000 TENS vs. SHAM: 1.000 PENS vs. TENS: 1.000

**GCPS**	Baseline	29.8 (19.73, 39.87)	30.8 (22.11, 39.49)	31.2 (25.81, 36.58)	15.22 (0.002)	11.33 (0.079)	
	Post-treat one-on-one	14.38 (9.15, 19.62)	22.63 (15.66, 29.59)	20.2 (15.15, 25.25)			PENS vs SHAM: 0.333 TENS vs. SHAM: 1.000 PENS vs. TENS: 0.168
	Post-treat at home	12.27 (5.73, 18.82)	25.00 (14.50, 35.50)	19.09 (10.21, 27.96)			PENS vs SHAM: 0.850 TENS vs. SHAM: 1.000 PENS vs. TENS: 0.254
	3 months follow-up	21.05 (11.94, 30.16)	16.06 (9.08, 23.03)	15.09 (7.00, 23.17)			PENS vs SHAM: 0.827 TENS vs. SHAM: 1.000 PENS vs. TENS: 0.888
**WOMAC**	Baseline	33.89 (21.15, 46.63)	37.86 (28.57, 47.16)	34.13 (26.17, 42.09)	31.44 (<0.001)	4.61 (0.595)	
	Post-treat one-on-one	17.03 (10.55, 23.52)	22.65 (13.75, 31.54)	17.21 (10.21, 24.21)			PENS vs SHAM: 1.000 TENS vs. SHAM: 1.000 PENS vs. TENS: 1.000
	Post-treat at home	20.17 (13.97, 26.37)	31.14 (18.43, 43.86)	17.52 (9.81, 25.24)			PENS vs SHAM: 1.000 TENS vs. SHAM: 0.258 PENS vs. TENS: 0.617
	3 months follow-up	17.93 (12.77, 23.09)	17.98 (7.89, 28.08)	15.08 (7.69, 22.48)			PENS vs SHAM: 1.000 TENS vs. SHAM: 1.000 PENS vs. TENS: 1.000
**TUG**	Baseline	9.09 (7.12, 11.06)	8.04 (6.54, 9.54)	9.17 (7.60, 10.74)	23.30 (<0.001)	8.80 (0.185)	
	Post-treat one-on-one	8.02 (6.50, 9.54)	7.86 (6.60, 9.13)	8.39 (7.08, 9.71)			PENS vs SHAM: 1.000 TENS vs. SHAM: 0.960 PENS vs. TENS: 0.867

	Post-treat at home	8.04 (6.51, 9.56)	7.48 (6.05, 8.90)	7.57 (6.52, 8.63)			PENS vs SHAM: 1.000 TENS vs. SHAM: 0.633 PENS vs. TENS: 1.000
	3 months follow-up	7.74 (6.62, 8.87)	7.13 (5.55, 8.72)	7.20 (6.10, 8.29)			PENS vs SHAM: 0.660 TENS vs. SHAM: 0.623 PENS vs. TENS: 1.000
**SPPB**	Baseline	8.5 (7.51, 9.49)	8.6 (7.70, 9.50)	8.39 (7.72, 9.05)	8.81 (0.012)	8.61 (0.197)	
	Post-treat one-on-one	9.05 (8.48, 9.63)	9.08 (8.17, 9.98)	8.94 (8.38, 9.50)			PENS vs SHAM: 1.000 TENS vs. SHAM: 1.000 PENS vs. TENS: 1.000
	Post-treat at home	9 (8.37, 9.63)	9.69 (8.92, 10.47)	9.4 (8.82, 9.98)			PENS vs SHAM: 1.000 TENS vs. SHAM: 1.000 PENS vs. TENS: 0.218
	3 months follow-up	9.1 (8.66, 9.54)	9.41 (8.51, 10.31)	9.4 (8.82, 9.98)			PENS vs SHAM: 1.000 TENS vs. SHAM: 1.000 PENS vs. TENS: 1.000

GCPS: Chronic Pain Grading Scale; PENS: Percutaneous electrical nerve stimulation; SPPB: Short Physical Performance Battery; TENS: Transcutaneous electrical nerve stimulation; TUG: Timed Up and Go; VAS: Visual analogue scale; WOMAC: Western Ontario and McMaster Universities Osteoarthritis Index questionnaire.

Table S4. Mixed-effects model results for pain sensitization measures

		**PENS** Margins (CI 95%)	**Control TENS** Margins (CI 95%)	**Sham PENS** Margins (CI 95%)	**Time factor** Chi² (p value)	**Group* Time factor** Chi² (p value)	**Pos-hoc comparisons** p value (Bonferroni)
**PPT** Symptomatic knee	Baseline	3.24 (2.38, 4.11)	3.66 (2.13, 5.19)	3.14 (2.04, 4.24)	6.21 (0.102)	1.84 (0.934)	
	Post-treat one-on-one	3.93 (3.10, 4.76)	4.37 (2.82, 5.92)	3.98 (2.91, 5.04)			PENS vs SHAM: 1.000 TENS vs. SHAM: 1.000 PENS vs. TENS: 1.000
	Post-treat at home	3.57 (2.86, 4.28)	4.50 (3.02, 5.98)	3.87 (3.14, 4.60)			PENS vs SHAM: 1.000 TENS vs. SHAM: 1.000 PENS vs. TENS: 0.508
	3 months follow-up	4.14 (3.34, 4.95)	4.97 (3.35, 6.59)	4.24 (3.33, 5.15)			PENS vs SHAM: 1.000 TENS vs. SHAM: 1.000 PENS vs. TENS: 1.000
**PPT** Tibialis anterior	Baseline	3.37 (2.57, 4.19)	3.42 (1.98, 4.87)	3.56 (2.12, 5.00)	6.09 (0.107)	3.81 (0.703)	
	Post-treat one-on-one	3.58 (2.83, 4.32)	4.28 (2.58, 6.00)	3.81 (3.00, 4.62)			PENS vs SHAM: 1.000 TENS vs. SHAM: 1.000 PENS vs. TENS: 0.993
	Post-treat at home	3.84 (2.83, 4.85)	4.00 (2.22, 5.78)	4.27 (3.44, 5.11)			PENS vs SHAM: 1.000 TENS vs. SHAM: 1.000 PENS vs. TENS: 1.000
	3 months follow-up	4.45 (3.64, 5.26)	4.60 (2.78, 6.41)	4.51 (3.73, 5.29)			PENS vs SHAM: 1.000 TENS vs. SHAM: 1.000 PENS vs. TENS: 1.000

**PPT** Asymptomatic knee	Baseline	3.75 (2.98, 4.52)	4.08 (2.56, 5.59)	4.03 (2.59, 5.47)	7.16 (0.067)	3.58 (0.747)	
	Post-treat one-on-one	3.71 (2.77, 4.66)	4.53 (3.09, 5.97)	4.06 (3.07, 5.07)			PENS vs SHAM: 1.000 TENS vs. SHAM: 1.000 PENS vs. TENS: 0.441
	Post-treat at home	3.79 (2.95, 4.63)	4.23 (2.67, 5.79)	4.20 (3.49, 4.90)			PENS vs SHAM: 1.000 TENS vs. SHAM: 1.000 PENS vs. TENS: 1.000
	3 months follow-up	4.71 (3.83, 5.59)	5.10 (3.45, 6.75)	4.77 (3.88, 5.67)			PENS vs SHAM: 1.000 TENS vs. SHAM: 1.000 PENS vs. TENS: 1.000
**PPT** Trapezius	Baseline	2.78 (2.13, 3.43)	2.84 (2.04, 3.64)	3.48 (2.23, 4.72)	2.64 (0.450)	7.21 (0.302)	
	Post-treat one-on-one	2.78 (2.19, 3.37)	3.22 (2.12, 4.31)	3.28 (2.51, 4.05)			PENS vs SHAM: 1.000 TENS vs. SHAM: 0.965 PENS vs. TENS: 1.000
	Post-treat at home	3.18 (2.45, 3.92)	3.73 (2.26, 5.20)	3.26 (2.57, 3.96)			PENS vs SHAM: 0.976 TENS vs. SHAM: 0.293 PENS vs. TENS: 1.000
	3 months follow-up	3.30 (2.87, 3.73)	4.20 (2.77, 5.62)	3.52 (2.91, 4.13)			PENS vs SHAM: 1.000 TENS vs. SHAM: 0.121 PENS vs. TENS: 0.189
**CPM**	Baseline	0.62 (-0.01, 1.26)	0.19 (-0.28, 0.66)	0.43 (0.08, 0.78)	8.77 (0.032)	5.97 (0.426)	
	Post-treat one-on-one	0.92 (0.49, 1.35)	0.75 (0.26, 1.24)	0.72 (0.32. 1.13)			PENS vs SHAM: 1.000 TENS vs. SHAM: 1.000 PENS vs. TENS: 1.000
	Post-treat at home	0.80 (0.35, 1.25)	0.25 (-0.24, 0.75)	0.89 (0.25, 1.52)			PENS vs SHAM: 1.000

							TENS vs. SHAM: 0.421 PENS vs. TENS: 0.530
	3 months follow-up	0.74 (0.17, 1.30)	0.99 (0.50, 1.49)	1.12 (0.73, 1.50)			PENS vs SHAM: 0.754 TENS vs. SHAM: 1.000 PENS vs. TENS: 0.884
**TSP**	Baseline	2.8 (1.31, 4.29)	2.7 (1.57, 3.83)	3.6 (2.70, 4.50)	53.11 (<0.001)	5.23 (0.514)	
	Post-treat one-on-one	2.5 (1.47, 3.53)	2.39 (1.34, 3.45)	2.1 (0.79, 3.40)			PENS vs SHAM: 0.921 TENS vs. SHAM: 0.927 PENS vs. TENS: 1.000
	Post-treat at home	2.3 (1.50, 3.10)	2.50 (1.35, 3.66)	2.2 (1.27, 3.13)			PENS vs SHAM: 0.544 TENS vs. SHAM: 0.625 PENS vs. TENS: 1.000
	3 months follow-up	1.5 (0.99, 2.01)	1.85 (0.60, 3.10)	1.6 (1.02, 2.18)			PENS vs SHAM: 1.000 TENS vs. SHAM: 1.000 PENS vs. TENS: 1.000

CPM: Conditioned pain modulation; PENS: Percutaneous electrical nerve stimulation; PPT: Pressure pain thresholds; TENS: Transcutaneous electrical nerve stimulation; TSP: Temporal summation of pain

Table S5. Mixed-effects model results for psychological outcomes

		**PENS** Margins (CI 95%)	**Control TENS** Margins (CI 95%)	**Sham PENS** Margins (CI 95%)	**Time factor** Chi^2 (p value)	**Group* Time factor** Chi^2 (p value)	**Pos-hoc comparisons** p value (Bonferroni)
**PCS**	Baseline	9.48 (3.69, 15.26)	14.77 (5.79, 23.74)	14.72 (9.47, 19.97)	41.48 (<0.001)	6.61 (0.359)	
	Post-treat one-on-one	2.57 (0.64, 4.50)	10.03 (3.31, 16.75)	6.73 (2.63, 10.83)			PENS vs SHAM: 0.332 TENS vs. SHAM: 1.000 PENS vs. TENS: 0.167
	Post-treat at home	5.05 (0.25, 9.86)	11.06 (2.08, 20.03)	4.53 (0.82, 8.25)			PENS vs SHAM: 0.922 TENS vs. SHAM: 0.389 PENS vs. TENS: 1.000
	3 months follow-up	3.20 (0.46, 5.95)	7.50 (0.53, 14.46)	5.19 (-0.13, 10.51)			PENS vs SHAM: 1.000 TENS vs. SHAM: 1.000 PENS vs. TENS: 1.000
**BDI-II**	Baseline	4.99 (2.22, 7.76)	5.00 (2.86, 7.13)	6.51 (3.32, 9.70)	11.23 (0.010)	5.80 (0.445)	
	Post-treat one-on-one	3.10 (175, 4.46)	4.13 (1.34, 6.92)	3.79 (1.72, 5.87)			PENS vs SHAM: 1.000 TENS vs. SHAM: 1.000 PENS vs. TENS: 1.000
	Post-treat at home	3.32 (1.59, 5.05)	4.80 (0.56, 0.05)	2.80 (1.37, 4.23)			PENS vs SHAM: 0.660 TENS vs. SHAM: 0.293 PENS vs. TENS: 1.000
	3 months follow-up	3.03 (1.19, 4.86)	5.24 (0.60, 9.88)	4.54 (1.45, 7.62)			PENS vs SHAM: 1.000 TENS vs. SHAM: 1.000 PENS vs. TENS: 1.000

**STAI**	Baseline	23.6 (21.19, 26.01)	22 (19.49, 24.50)	22.6 (20.43, 24.77)	0.29 (0.961)	5.42 (0.491)	
	Post-treat one-on-one	21.99 (19.59, 24.40)	23.16 (20.52, 25.80)	22.38 (19.66, 25.10)			PENS vs SHAM: 0.835 TENS vs. SHAM: 1.000 PENS vs. TENS: 0.324
	Post-treat at home	22.11 (19.31, 24.91)	22.73 (19.83, 25.63)	22.14 (20.17, 24.11)			PENS vs SHAM: 1.000 TENS vs. SHAM: 1.000 PENS vs. TENS: 0.613
	3 months follow-up	24.91 (22.76, 27.06)	23.81 (21.36, 26.27)	21.90 (18.99, 24.80)			PENS vs SHAM: 0.369 TENS vs. SHAM: 0.674 PENS vs. TENS: 1.000
**TSK-11**	Baseline	28.7 (25.40, 32.00)	30.2 (25.36, 35.04)	27.2 (24.26, 30.14)	5.45 (0.141)	9.40 (0.152)	
	Post-treat one-on-one	20.06 (17.63, 22.49)	25.19 (20.85, 29.53)	24.8 (21.70, 27.90)			PENS vs SHAM: 0.030 TENS vs. SHAM: 1.000 PENS vs. TENS: 0.169
	Post-treat at home	22.73 (19.48, 25.98)	23.80 (17.63, 29.96)	23.17 (19.98, 26.35)			PENS vs SHAM: 1.000 TENS vs. SHAM: 1.000 PENS vs. TENS: 1.000
	3 months follow-up	19.37 (15.80, 22.95)	21.12 (15.86, 26.38)	21.94 (18.61, 25.28)			PENS vs SHAM: 0.671 TENS vs. SHAM: 1.000 PENS vs. TENS: 1.000

BDI-II: Beck Depression Inventory; PCS: Pain Catastrophizing Scale; PENS: Percutaneous electrical nerve stimulation; STAI: State Anxiety Inventory; TENS: Transcutaneous electrical nerve stimulation; TSK-11: Tampa Scale for Kinesiophobia.
